# Associations of retinal microvascular alterations with diabetes mellitus: an OCTA-based cross-sectional study

**DOI:** 10.1186/s12886-024-03492-9

**Published:** 2024-06-10

**Authors:** Yao Yao, Qian Wang, Jingyan Yang, Yanni Yan, Wenbin Wei

**Affiliations:** Laboratory of Intraocular Tumor Diagnosis and Treatment, Ophthalmology&Visual Sciences Key Lab, Medical Artificial Intelligence Research and Verification Key Laboratory of the Ministry of Industry and Information Technology, Beijing Tongren Eye Center, Beijing Tongren Hospital, Capital Medical University, Beijingkey, 100730 China

**Keywords:** Optical coherence tomography angiography (OCTA), Retinal microvasculature, Capillary density, Diabetes mellitus, Hypertension

## Abstract

**Background:**

Diabetes, a health crisis afflicting millions worldwide, is increasing rapidly in prevalence. The microvascular complications triggered by diabetes have emerged as the principal cause of renal disease and blindness. The retinal microvascular network may be sensitive to early systemic vascular structural and functional changes. Therefore, this research endeavored to discern the systemic determinants influencing the retinal microvascular network in patients with and without diabetes.

**Methods:**

The Kailuan Eye Study is a cross-sectional study based on the community-based cohort Kailuan Study. Participants underwent optical coherence tomography angiography (OCTA) (Zeiss Cirrus 5000; Carl Zeiss Meditec) and comprehensive systemic examination. Metrics such as perfusion density (PD), vascular density (VD), foveal avascular zone (FAZ) parameters of the superficial capillary plexus (SCP) in the macula were assessed.

**Results:**

This study included 860 eligible participants (average age = 62.75 ± 6.52 years; 21.9% female), of which 449 were diabetics. People with diabetes had diminished PD and VD in the entire macular and parafoveal regions compared to people without diabetes. Reduced PD in the whole macular region was correlated with higher fasting plasma glucose (FPG, mmol/L) concentration (Beta = -0.19, 95% CI = -0.42 to -0.36, *P* < 0.001), longer axial length (AL, mm) (Beta = -0.13, 95%CI = -0.48 to -0.25, *P* = 0.002), and elevated heart rate (Beta = -0.10, 95%CI = -0.14 to -0.19, *P* = 0.014), after adjusting for younger age (Beta = -0.18, 95%CI = -0.24 to -0.35, *P* < 0.001), consistent with VD of the whole macular region. A higher FPG level was significantly correlated with lower SCP density of both PD and VD in the macular and parafoveal region (*P* < 0.05 for all), as well as increased systolic blood pressure and low-density lipoprotein cholesterol concentration (*P* < 0.01 for all).

**Conclusions:**

In this large-sample cross-sectional study, OCTA evaluation revealed that high prevalence of diabetes and elevated FPG levels were correlated with reduced retinal VD and PD. Hypertension and hyperlipidemia are important risk factors for the development of atherosclerotic cardiovascular disease but have no significant effect on retinal microvascular abnormalities.

## Background

Diabetes, a global health crisis of the twenty-first century, currently plagues millions of individuals across the globe [1]. In developed nations, diabetes-induced microvascular complications are the primary cause of blindness and end-stage renal disease [2]. Studies utilizing OCTA consistently report a reduction in vessel density, particularly in the superficial and deep capillary plexuses, in diabetic patients compared to healthy controls [3]. Decreased vessel density is indicative of microvascular dropout and impaired perfusion in diabetic retinopathy [4]. Decreased perfusion density reflects compromised blood flow in the retinal microvasculature, which is associated with disease severity and progression, highlighting its potential as a biomarker for monitoring diabetic retinopathy.

The retinal vascular network plays a crucial role in preserving optimal retinal functionality. A comprehensive evaluation of the retinal vascular system’s structure and function is indispensable for the diagnosis, therapeutic intervention, and management of numerous retinal pathologies.Alterations in retinal vessels represent cumulative responses to aging, cardiovascular risk factors, inflammation, endothelial dysfunction, among other elements [5–7]. Early detection of microvascular modifications can thus serve as a potent screening tool, particularly for identifying both ocular and systemic complications of cardiovascular disease in their early stages. The advent of optical coherence tomography angiography (OCTA) offers a methodology to quantify capillary blood flow and map of perfused blood vessels [8]. Utilizing OCTA allows for the automatic segmentation and visualization of specific layers of the capillary plexus, thus it is considered as an innovative instrument for evaluating the microvascular system in systemic diseases [9, 10].

Although there have been many studies on retinal microvascular changes in the Chinese diabetic population, there is limited evidence from a large-sample cohort regarding the correlation between multiple systemic risk factors and retinal microvascular impairment. Large-sample study allows for sufficient statistical power to detect associations between systemic risk factors and retinal microvascular impairment, even after adjusting for potential confounders. Therefore, this study endeavors to assess the distribution of retinal microvascular parameters and their potential correlation with systemic risk factors such as age, sex, blood pressure, glucose, and lipids in a population including people without or with only mild diabetic retinopathy. Furthermore, it aims to investigate whether retinal microvascular parameters can serve as a reliable indicator for systemic disease assessment.

## Methods

### Study population

**Fig. 1:**
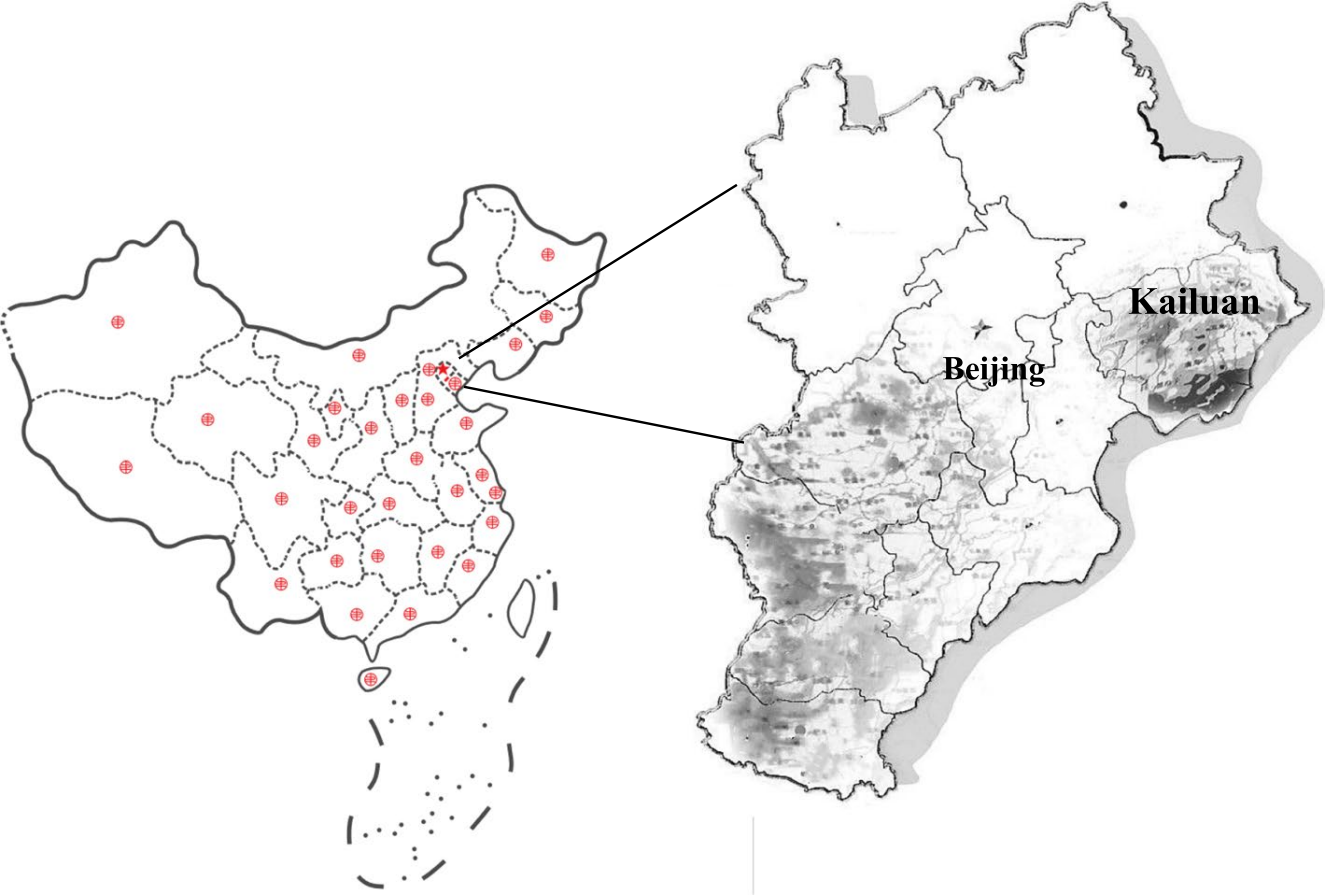
Graphical representation of the location for the study in China

The Kailuan Eye Study, a cross-sectional investigation, incorporated participants from a longitudinal community-based cohort study. Initially, between 2006 and 2007, the cohort included 101,510 participants from the Kailuan community, aged between 18 and 98 years, with assessments repeated biennially. Based on a unit-based cluster random sampling method, 14,440 individuals were randomly selected from the Kailuan cohort and consented to participate in the Kailuan Eye Study (Fig. 1). All participants underwent standardized interviews and laboratory assessments at each interval. From March to June 2017, 897 eligible applicants were randomly recruited through voluntary enrollment and screening and underwent OCTA examination additionally. Subjects were included without considering age, gender, and other systemic medical history, except for exclusion criteria. Exclusion criteria encompassed a history of ocular diseases (excluding age-related cataract), previous intraocular surgery (excluding cataract or refractive surgeries), scans with low signal strength (OCTA Scan Quality Index < 6), or blurred images. Participants with myopia, incipient cataract, or pseudophakia that did not interfere with OCTA imaging qualities were not excluded. The Kailuan Eye Study received approval from the Medical Ethics Committee of the Kailuan General Hospital, Beijing Tongren Hospital, and Peking University First Hospital, adhering to the tenets of the Declaration of Helsinki. All participants provided written informed consent.

### Assessment of systemic risk factors

Clinical data and personal information were collected from all participants by trained interviewers through a standardized and systematic examination. Face-to-face questionnaires were administered by research physicians. The collected information included demographic and socioeconomic data, lifestyle habits (including smoking, alcohol consumption, and exercise), and self-reported medical history (such as diabetes, hypertension, dyslipidemia, cardiovascular disease, thyroid diseases, stroke, family history, current medication intake, etc.). Blood pressure (BP) was measured using a mercury sphygmomanometer following standard procedures. Two readings were taken at a 5-min interval after participants had been seated for at least 5 min, with the average recorded for data analysis. Body weight, height, waist and hip circumferences (WC and HC) were measured directly. The body mass index (BMI) was calculated as weight in kilograms divided by height in metres squared. Blood samples were collected from all participants under overnight fasting conditions to test fasting plasma glucose (FPG), high-density lipoprotein cholesterol (HDL-C), low-density lipoprotein cholesterol (LDL-C), triglycerides (TG), total cholesterol (TC), uric acid (UA), hypersensitive C-reactive protein (hsCRP), glutamate pyruvate transaminase (GPT), total serum bilirubin, and serum creatinine at the clinical laboratory of the Kailuan General Hospital. The diagnostic criteria for diabetes included an FPG concentration ≥ 7.0 mmol/l during the 10-year follow-up period, a self-reported history of diabetes or a history of taking hypoglycaemic agents.

### Ocular examinations and OCTA parameters

Ocular examinations included measurements of visual acuity (VA), tonometry, slit-lamp assisted biomicroscopy of the anterior segment, and ocular biometry, which included central corneal thickness, corneal curvature, anterior chamber depth (ACD), lens thickness (LT), and axial length (AL) using optical low-coherence reflectometry (Lenstar 900 Optical Biometer; Haag-Streit, Koeniz, Switzerland), and optical coherence tomography angiography (OCTA) (Zeiss Cirrus 5000; Carl Zeiss Meditec, Dublin, CA, USA). Two 45° fundus photographs were obtained on the optic nerve head and macula using a nonmydriatic fundus camera (CR6-45NM; Canon, Inc., Osta, Tokyo, Japan). Pupils were medically dilated using eye drops containing 0.5% tropicamide and 0.5% phenylephrine hydrochloride.

**Fig. 2:**
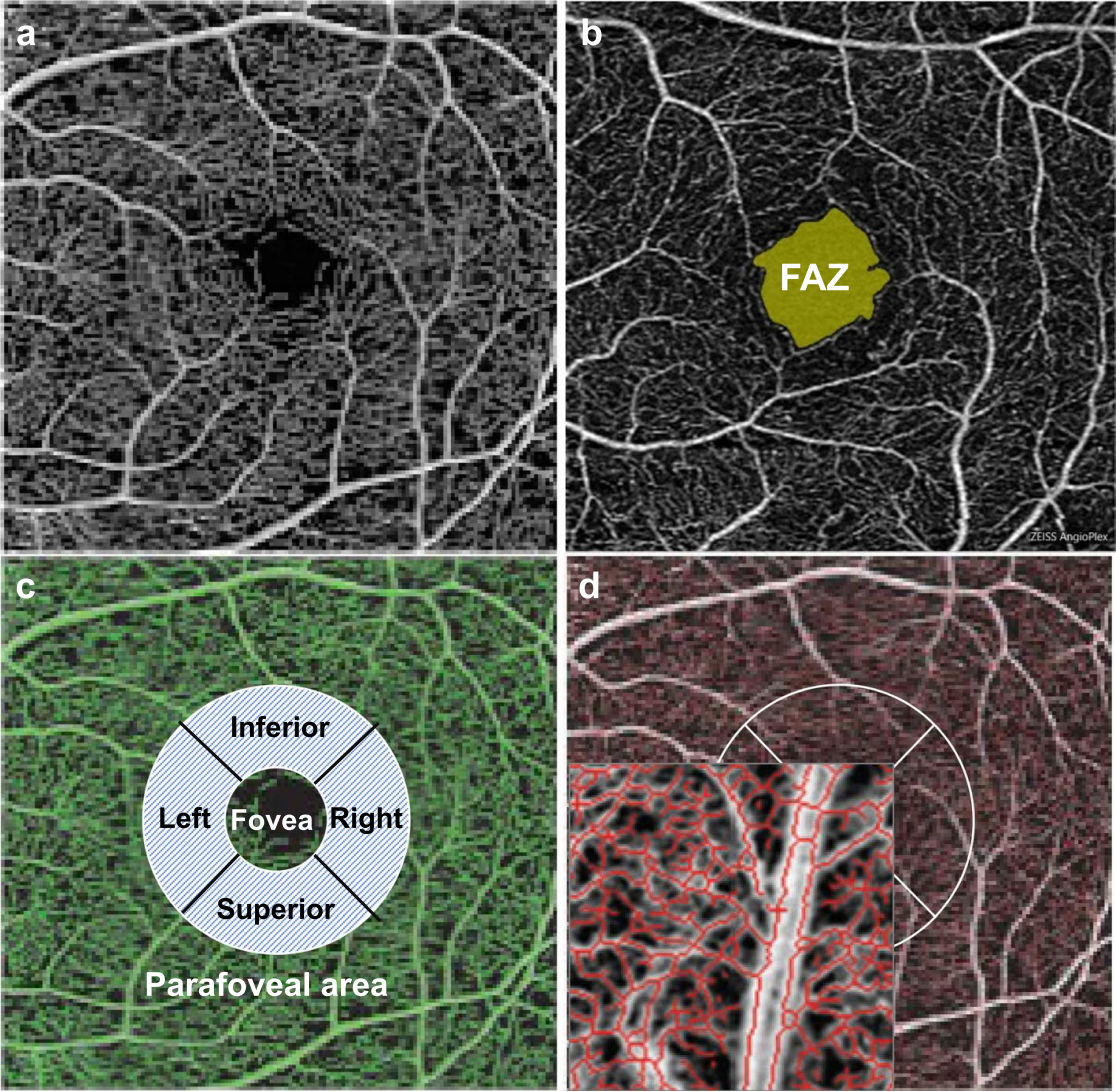
Representative OCTA images obtained by Zeiss Cirrus 5000 device (AngioPlex Metrix version 10.0). **a–d** Macula was screened by angio retina modes ( 6 × 6 mm). **a** OCTA image of SCP, defined as ILM to IPL -10 μm. **b** The FAZ area was automatically detected on the Retina slab. **c** Perfusion density and vascular density was automatically measured in SCP. Macula was regionally measured in the whole ETDRS grid area, which comprised 2 concentric rings: 1 mm fovea center, 1 to -3 mm parafoveal area and the whole 6 × 6mm area. **d** The vessel density (VD) (mm^−1^) was defined as the total length of perfused vasculature per unit area in the region of measurement

A 6mm × 6mm OCTA scan of the macula centered in the foveal area was performed using a Zeiss Cirrus 5000 machine (Carl Zeiss Meditec, USA). Qualified images met the following criteria: signal strength ≥ 6, no more than one blink artifact or poor fixation leading to motion artifact, no refractive interstitial clouding, and a centered retinal vascular system and scan image. Vascular images of the superficial capillary plexus (SCP) from the internal limiting membrane to the inner plexiform layer were automatically displayed. All scans were analyzed using Cirrus OCTA software (AngioPlex Metrix version 10.0), and the measurement region of the scan was divided into three subregions: a 1mm diameter circle (foveal area), a 3mm diameter annulus outside the fovea (the parafoveal area), and the entire 6mm x 6mm macular area (Fig. 2). These were identical to the Early Treatment Diabetic Retinopathy Study (ETDRs) subregion. Perfusion density (PD) and vascular density (VD) of the SCP were assessed automatically. VD, quantified in mm^−1^, is delineated as the aggregate length of perfused vasculature within a designated measurement region, while PD signifies the total area of the perfused vascular network within the identical unit area. We conducted a separate evaluation of VD and PD in the SCP located in the foveal area, the parafoveal area, and the entire macular region. The foveal avascular zone (FAZ) was automatically detected on the retina slab (ILM to OPL + 10μm). The FAZ circularity index was defined as the ratio between the measured perimeter and the perimeter of an equivalent circular area. Both fundus images and OCTA scans were evaluated by experienced and trained ophthalmologists (Y.Y and Q.W.). In case of uncertainty, images were reassessed by several ophthalmologists (Q.W., Y.X.W., Y.N.Y., and W.B.W.). Both eyes were examined but only data from right eyes were included in the analysis.

### Statistical analysis

Statistical analyses were conducted utilizing a commercially available statistical software program (SPSS, version 27.0; IBM/SPSS, Chicago, IL, USA). Continuous variables were articulated as either mean ± standard deviation or median (quartiles), while categorical variables were expressed as number (proportions). Independent sample t-test was used to compare the systemic and retinal microvascular parameters between diabetics and non-diabetics. The relationship between systemic factors and OCTA parameters was evaluated using univariate linear regression analysis. To identify statistically significant variables for retinal microvascular parameters, multivariate linear regression models were employed. A two-tailed P value less than 0.05 was established as statistically significant at the 95% CI level.

## Results

**Fig. 3:**
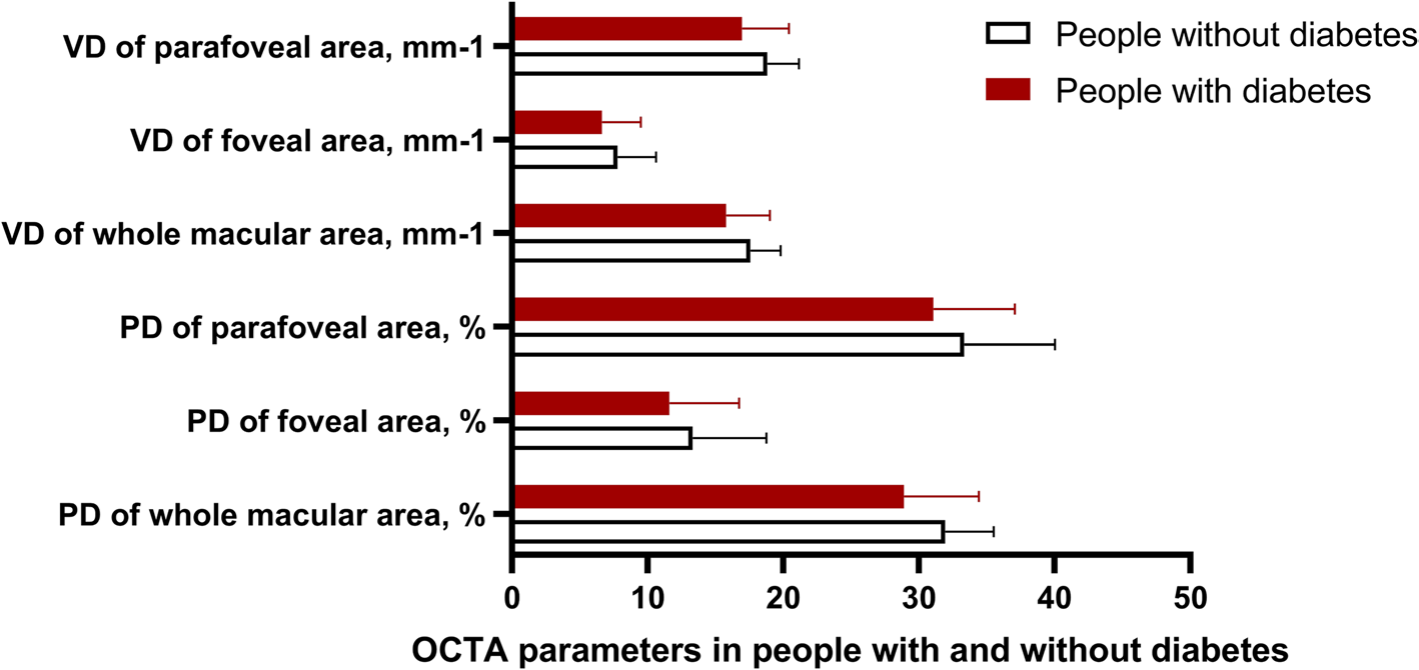
PD, perfusion density; VD, vessel density; OCTA, optical coherence tomography angiography. The mean retinal perfusion and vessel density in people without diabetes were significantly higher than in people with diabetes. (Table 1)

A total of 897 participants underwent the OCTA examination. However, based on the exclusion criteria, 21 participants with prior ocular diseases or surgeries, and 16 participants with low OCTA scan signals or unreadable images were excluded. Consequently, 860 eligible individuals (188 females, representing 21.9%) were included in the statistical analysis of this study, with ages ranging from 27 to 83 years (Mean age = 62.75 ± 6.52 years). The cohort included 449 people in the diabetes group and 411 people in the non-diabetic group, with no significant age or sex differences between the two groups. The systemic and ocular variables of DM and non-DM groups are detailed in Table 1. Independent sample t-tests revealed that diabetic people had significantly higher systolic blood pressure (SBP), BMI, WC, heart rate, LDL-C, TG, TC, UA, hsCRP, GPT, FPG and total bilirubin concentrations, and lower HDL-C concentrations compared to non-diabetic people (Table 1, all *P* < 0.05). In terms of OCTA parameters, diabetic people had lower PD and VD in all measured regions (Fig. 3) and a smaller FAZ area and lower FAZ circularity than non-diabetic people.

**Table 1:** Descriptive information of the systemic and ocular variable

	DM group		non-DM group		*P*-value
	Mean	SD	Mean	SD	
Age, years	62.34	7.39	63.16	5.47	0.07
Anterior chamber depth, mm	2.62	0.44	2.70	0.40	< 0.001
lens thickness, mm	4.45	0.68	4.48	0.34	0.761
Axial length, mm	23.43	0.94	23.45	1.03	0.311
Systolic blood pressure,mmHg	145.05	18.72	133.32	16.10	< 0.001
Diastolic blood pressure,mmHg	83.02	10.15	83.56	8.25	0.288
Height, m	168.78	6.47	168.21	6.49	0.247
Weight, kg	73.11	10.56	71.10	9.87	0.010
Body Mass Index, kg/m^2^	25.91	3.52	25.09	2.92	< 0.001
Waist circumference, cm	89.52	9.13	87.41	7.49	< 0.001
Hip circumference, cm	96.38	10.17	97.59	6.70	0.063
Heart rate	77.31	11.43	71.24	8.66	< 0.001
Fasting plasma glucose, mmol/L	8.09	2.24	5.30	0.43	< 0.001
High-density lipoprotein cholesterol, mmol/L	1.45	0.34	1.60	0.34	< 0.001
Low-density lipoprotein cholesterol, mmol/L	2.96	0.78	2.55	0.68	< 0.001
Triglyceride, mmol/L	2.09	2.33	1.67	1.94	0.017
Total cholesterol, mmol/L	5.53	1.07	5.23	0.93	< 0.001
Uric acid, mmol/L	346.79	80.55	328.78	78.46	0.003
Hypersensitive C-reactive protein, mmol/L	2.79	3.51	2.30	2.82	0.027
Glutamate pyruvate transaminase, mmol/L	22.90	14.57	20.77	11.37	0.046
Total bilirubin, mmol/L	16.43	6.41	15.11	4.64	0.003
Creatinine, mmol/L	76.89	65.50	77.79	23.16	0.917
Signal strength of scan	8.11	1.32	8.76	1.06	< 0.001
FAZ area, mm^2^	0.32	0.12	0.28	0.15	< 0.001
FAZ boundary, mm	2.26	0.87	2.55	0.66	< 0.001
FAZ circularity, %	0.57	0.19	0.09	0.22	< 0.001
PD of whole area, %	28.91	5.49	31.93	3.58	< 0.001
PD of foveal area, %	11.62	5.13	13.32	5.44	< 0.001
PD of parafoveal area, %	31.06	6.01	33.35	6.66	< 0.001
PD of the parafoveal right area, %	30.41	6.86	32.70	4.80	< 0.001
PD of the parafoveal superior area, %	31.34	6.53	34.72	4.77	< 0.001
PD of the parafoveal left area, %	31.77	6.76	35.18	4.55	< 0.001
PD of the parafoveal inferior area, %	30.96	7.14	34.40	4.36	< 0.001
VD of whole area, mm^−1^	15.79	3.23	17.59	2.20	< 0.001
VD of foveal area, mm^−1^	6.64	2.86	7.79	2.83	< 0.001
VD of parafoveal area, mm^−1^	16.97	3.43	18.84	2.31	< 0.001
VD of the parafoveal right area, mm^−1^	16.50	3.75	17.96	2.67	< 0.001
VD of the parafoveal superior area, mm^−1^	17.06	3.74	19.02	2.72	< 0.001
VD of the parafoveal left area, mm^−1^	17.32	3.72	19.28	2.57	< 0.001
VD of the parafoveal inferior area, mm^−1^	17.01	4.06	19.09	2.59	< 0.001

*FAZ* Foveal avascular zone, *PD* Perfusion density, *VD* Vascular density

In the analysis involving all participants, univariate linear regression analysis with ‘DM status’ as a covariate demonstrated that VD and PD of the whole 6mm x 6mm area, foveal area and parafoveal area were all correlated with age and FPG (all *P* < 0.05) (Table 2). Additionally, PD and VD of the whole macular region were also correlated with SBP, heart rate, and AL (all *P* < 0.05). VD of the foveal region was correlated with ACD, LT and AL (all *P* < 0.05).

**Table 2:** Univariate linear regression analysis between retinal microvascular and systemic variables in all participants

Variates	PD of whole area		PD of fovea		PD of parafoveal area		VD of whole area		VD of fovea		VD of parafoveal area	
	Beta	P value	Beta	P value	Beta	P value	Beta	P value	Beta	P value	Beta	P value
Age	-0.21	** < 0.001**	-0.08	0.01	-0.14	** < 0.001**	-0.23	** < 0.001**	-0.12	** < 0.001**	-0.23	** < 0.001**
Gender	0.04	0.28	-0.03	0.44	0.02	0.62	0.05	0.15	-0.02	0.64	0.05	0.11
Systolic blood pressure	-0.10	0.004	-0.05	0.14	-0.09	0.01	-0.11	0.001	-0.06	0.08	-0.11	0.001
Diastolic blood pressure	0.00	1.00	-0.02	0.59	-0.03	0.43	0.00	0.92	-0.01	0.84	0.00	0.91
Height	-0.05	0.15	0.02	0.67	-0.05	0.21	-0.06	0.13	0.02	0.67	-0.06	0.09
Weight	-0.03	0.50	-0.01	0.73	-0.04	0.25	-0.03	0.36	-0.01	0.91	-0.04	0.33
BMI	-0.03	0.36	-0.03	0.41	-0.05	0.19	-0.04	0.25	-0.02	0.48	-0.04	0.26
Waist circumference	-0.04	0.23	-0.01	0.75	-0.05	0.14	-0.06	0.11	-0.02	0.61	-0.06	0.09
Hip circumference	0.01	0.70	0.01	0.71	0.00	0.93	0.01	0.88	0.01	0.77	0.00	0.92
Heart rate	-0.08	0.047	-0.12	0.004	-0.04	0.39	-0.09	0.045	-0.14	0.001	-0.07	0.09
Fasting plasma glucose	-0.31	** < 0.001**	-0.15	** < 0.001**	-0.23	** < 0.001**	-0.31	** < 0.001**	-0.16	** < 0.001**	-0.31	** < 0.001**
High-density lipoprotein cholesterol	-0.02	0.58	-0.01	0.78	-0.02	0.52	-0.02	0.59	0.00	0.90	-0.02	0.58
Low-density lipoprotein cholesterol	0.02	0.51	-0.01	0.73	0.03	0.42	0.02	0.52	-0.01	0.71	0.03	0.46
Triglyceride	0.06	0.06	0.00	0.95	0.06	0.09	0.07	0.05	0.00	0.92	0.07	0.04
Total cholesterol	0.00	0.91	0.00	0.91	-0.03	0.44	0.01	0.82	0.02	0.64	0.01	0.85
Uric acid	-0.02	0.65	0.05	0.17	-0.02	0.48	-0.02	0.54	0.05	0.14	-0.03	0.41
Hypersensitive C-reactive protein	-0.03	0.33	-0.02	0.56	-0.02	0.50	-0.04	0.26	-0.02	0.49	-0.04	0.26
Glutamate pyruvate transaminase	0.04	0.26	0.01	0.73	0.03	0.41	0.04	0.31	0.01	0.78	0.04	0.29
Total bilirubin	0.00	0.95	0.02	0.63	0.00	0.97	-0.01	0.84	0.02	0.68	-0.01	0.79
Creatinine	0.01	0.71	-0.05	0.19	0.02	0.56	0.01	0.71	-0.05	0.16	0.02	0.58
Anterior chamber depth	0.05	0.14	0.10	0.003	-0.01	0.72	0.06	0.07	0.14	** < 0.001**	0.05	0.16
Lens thickness	-0.05	0.15	-0.05	0.17	0.00	0.98	-0.05	0.13	-0.08	0.03	-0.05	0.17
Axial length	-0.11	0.001	0.06	0.08	-0.13	** < 0.001**	-0.10	0.004	0.10	0.01	-0.12	** < 0.001**

Statistically significant parameters were uniformly bolded when *P* < 0.05

The multivariate linear regression model included OCTA parameters as dependent variables and those variables that were correlated with OCTA parameters with a P-value ≤ 0.10 (Table 2) as independent parameters. Variables showing covariance between variables (including ACD, LT, WC, Weight) or poor correlation with the dependent variable were excluded (Table 3). In the final model, higher PD in the whole macular region was correlated with lower FPG concentration (Beta = -0.19, *P* < 0.001), shorter AL (Beta = -0.13, *P* = 0.002), and slower heart rate (Beta = -0.10, *P* = 0.014), after adjusting for younger age (Beta = -0.18, *P* < 0.001), consistent with VD of the whole macular region. In the parafoveal region, higher PD and VD were both correlated with lower FPG concentration (Beta = -0.20 for PD and -0.19 for VD, both *P* < 0.001), shorter AL ( Beta = -0.12 for PD and -0.14 for VD, both *P* = 0.001) and younger age (*P* < 0.05 for all). Higher PD of fovea was only correlated with lower heart rate ( Beta = -0.13, *P* = 0.003), but VD showed correlation with longer AL, and lower heart rate, after adjusting for younger age ( Beta = -0.11, *P* = 0.008) and lower FPG ( Beta = -0.08, *P* = 0.053). The association between OCTA and systemic parameters was compared in both diabetic and non-diabetic groups. In people with diabetes, PD and VD in the whole region were negatively correlated FPG only ( Beta = 0.14, *P* = 0.022 for PD; Beta = 0.13, *P* = 0.046 for VD) after adjusting for age. Conversely, in non-diabetes people, higher PD and VD in the whole region were correlated with higher LDL-C ( Beta = 0.14, *P* = 0.022 for PD; Beta = 0.13, *P = *0.034 for VD) and total serum bilirubin concentration ( Beta = 0.14, *P *= 0.002 for PD; Beta = 0.15, *P* = 0.005 for VD), adjusted for AL and age.

**Table 3:** Multivariate linear regression model of OCTA and systemic parameters in all participants (only statistically significant parameters displayed)

Parameters	Standardized coeffecient	95%CI			*P* value	VIF
PD of whole area						
Fasting plasma glucose	-0.19	-0.42	,	-0.36	< 0.001	1.12
Age	-0.18	-0.24	,	-0.35	< 0.001	1.09
Axial length	-0.13	-0.48	,	-0.25	0.002	1.01
Heart rate	-0.1	-0.14	,	-0.19	0.014	1.12
PD of parafoveal area						
Axial length	-0.12	-0.61	,	-0.24	0.001	1.01
Fasting plasma glucose	-0.2	-0.45	,	-0.39	< 0.001	1.1
Age	-0.1	-0.18	,	-0.18	0.011	1.07
PD of foveal area						
Heart rate	-0.13	-0.17	,	-0.25	0.003	1.08
VD of whole area						
Fasting plasma glucose	-0.19	-0.33	,	-0.37	< 0.001	1.12
Age	-0.2	-0.24	,	-0.39	< 0.001	1.09
Axial length	-0.11	-0.32	,	-0.20	0.008	1.01
Heart rate	-0.1	-0.12	,	-0.18	0.017	1.12
VD of parafoveal area						
Fasting plasma glucose	-0.19	-0.33	,	-0.37	< 0.001	1.13
Age	-0.18	-0.22	,	-0.36	< 0.001	1.12
Axial length	-0.14	-0.37	,	-0.28	0.001	1.05
Heart rate	-0.1	-0.12	,	-0.16	0.024	1.12
VD of foveal area						
Axial length	0.12	-0.12	,	0.24	0.004	1.01
Heart rate	-0.15	-0.17	,	-0.29	0.001	1.11
Age	-0.11	-0.16	,	-0.22	0.008	1.09

*PD* Perfusion density, *VD* Vascular density

**Fig. 4:**
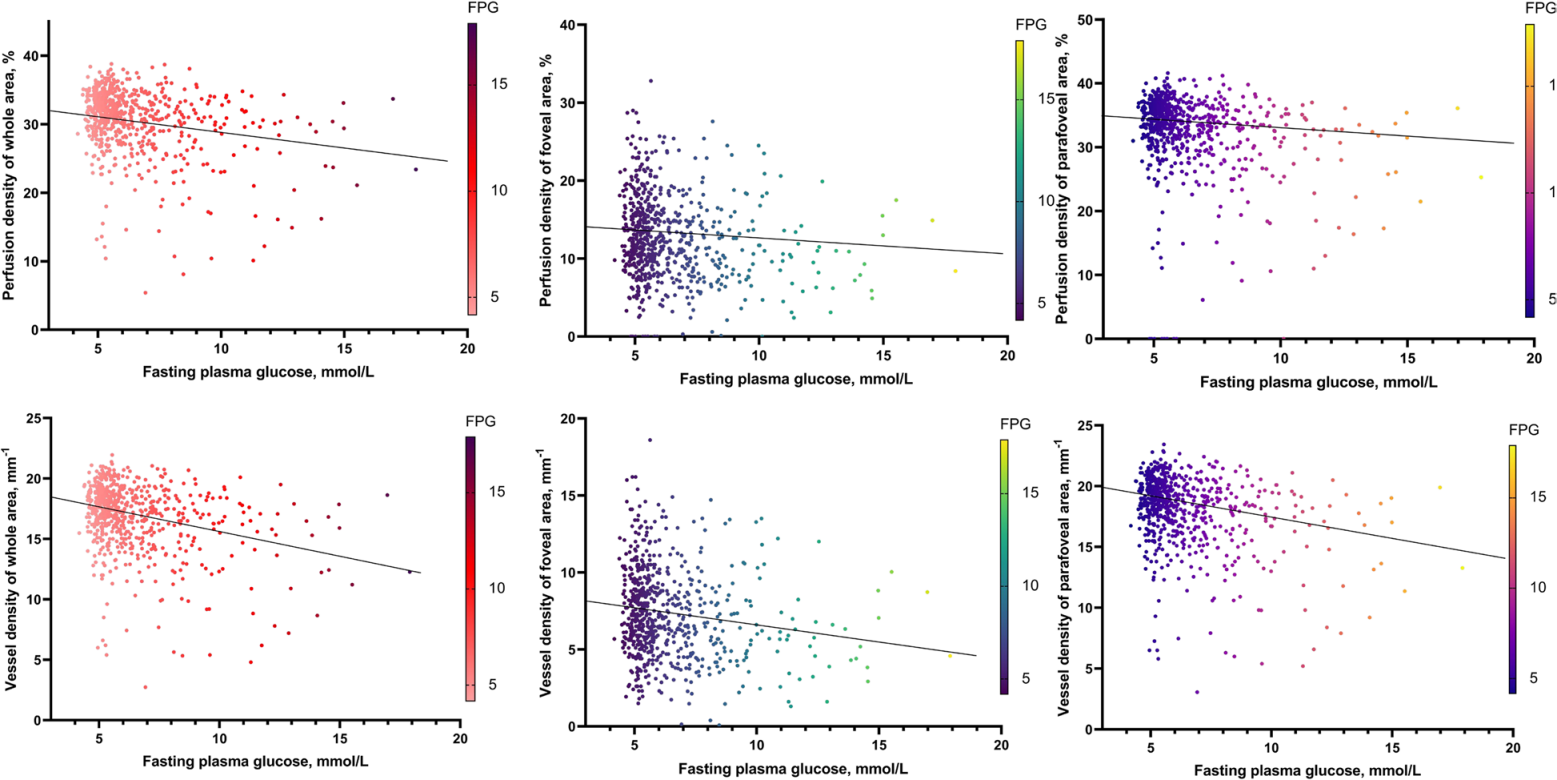
Scatter plots showing relationships between FPG and OCTA parameters. Solid lines inside the plots represent the univariate linear models

Multivariable linear regression model was also constructed between FPG and ocular parameters (Fig. 4), adjusting for confounding systemic factors. A higher FPG concentration was significantly correlated with lower SCP density of both PD and VD in the macular (Beta = -0.18, *P* < 0.001 for PD; Beta = -0.18, *P* < 0.001 for VD) and parafoveal regions (Beta = -0.11, *P* = 0.007 for PD; Beta = -0.18, *P* < 0.001 for VD). Moreover, higher FPG concentration was also assciated with higher SBP, heart rate, concentration of TG and LDL-C (*P* < 0.05 for all). (Table 4).

**Table 4:** Multivariable linear regression model between fasting plasma glucose and OCTA and systemic parameters

Parameters	Standardized coeffecient	95%CI			P value	VIF
When PD of whole area added in the model						
Heart rate	0.03	0.16	,	0.18	< 0.001	1.09
Systolic blood pressure	0.01	0.14	,	0.16	< 0.001	1.10
Perfusion density of whole area	-0.07	-0.21	,	-0.15	< 0.001	1.04
Low-density lipoprotein cholesterol	0.29	-0.04	,	0.30	0.001	1.03
Triglyceride	0.06	0.02	,	0.14	0.046	1.04
When PD of parafoveal area added in the model						
Heart rate	0.03	0.19	,	0.21	< 0.001	1.06
Systolic blood pressure	0.02	0.16	,	0.17	< 0.001	1.08
Perfusion density of parafoveal area	-0.03	-0.04	,	0.30	0.007	1.03
Low-density lipoprotein cholesterol	-0.03	-0.12	,	-0.08	0.010	1.02
When PD of foveal area added in the model						
Heart rate	0.03	0.19	,	0.22	< 0.001	1.06
Systolic blood pressure	0.02	0.17	,	0.18	< 0.001	1.07
Low-density lipoprotein cholesterol	0.27	-0.05	,	0.30	0.002	1.02
When VD of whole area added in the model						
Heart rate	0.03	0.16	,	0.18	< 0.001	1.09
Systolic blood pressure	0.01	0.14	,	0.15	< 0.001	1.10
Vascular density of whole area	-0.11	-0.23	,	-0.13	< 0.001	1.05
Low-density lipoprotein cholesterol	0.29	-0.04	,	0.30	0.001	1.03
Triglyceride	0.06	0.02	,	0.14	0.045	1.04
When VD of parafoveal area added in the model						
Heart rate	0.03	0.16	,	0.19	< 0.001	1.09
Vascular density of parafoveal area	-0.11	-0.23	,	-0.14	< 0.001	1.04
Systolic blood pressure	0.01	0.14	,	0.15	< 0.001	1.10
Low-density lipoprotein cholesterol	0.30	-0.04	,	0.30	0.001	1.03
Triglyceride	0.06	0.02	,	0.14	0.043	1.04
When VD of foveal area added in the model						
Heart rate	0.03	0.19	,	0.21	< 0.001	1.06
Systolic blood pressure	0.02	0.17	,	0.19	< 0.001	1.07
Low-density lipoprotein cholesterol	0.28	-0.05	,	0.30	0.002	1.02

*PD* Perfusion density, *VD* Vascular density

## Discussion

As a highly vascularized tissue, the retina is susceptible to microvascular damage, and retinal imaging provides a non-invasive method for detecting minor changes. Microvascular conditions in retinal vessels often reflect systemic or cardiovascular conditions due to their similar vascular scale and pathology. In this study, we conducted a cross-sectional analysis of OCTA images obtained from a cohort of 449 diabetes people with either no DR or mild NPDR. This cohort, compared to controls, exhibited a decreased macular PD and macular and parafoveal VD in the SCP. Given that this diabetes cohort was limited to eyes with no or mild signs of diabetic retinopathy, these findings suggest that a decrease in macular and parafoveal capillary density is an early process in the disease and initially occurs at the level of the SCP. The decrease in parafoveal vessel density is likely a result of diffuse capillary loss or nonperfusion, rather than FAZ enlargement or remodelling.

This study discovered that blood pressure, BMI, WC, HR, blood concentration of LDL-C, TG, TC, UA, hsCRP, GPT, FPG and total bilirubin were higher in diabetic people compared to the non-diabetes group. People with diabetes may be more prone to hypertension and hyperlipidemia, not only because there is a significant overlap in the population with a high prevalence of diabetes and hypertension and hyperlipidemia (poor diet and exercise habits, etc.), but also because changes in hemodynamic parameters due to elevated blood glucose are more likely to trigger elevated blood pressure and lipids [11–13]. In addition, the role of inflammation in the development of diabetes has long been established, and levels of inflammatory factors, including CRP and tumour necrosis factor-α, are often elevated in diabetic people [14, 15]. Similarly, elevated GPT activity has been confirmed to increase the risk of diabetes [16, 17]. This is generally consistent with the findings of the present study.

Intriguingly, despite the inclusion of a multitude of systemic factors in our study, only FPG level was found to be significantly associated with retinal microvascular parameters after adjusting for age and AL. This was also observed when the diabetes group was analyzed separately, with FPG levels showing a significant and negative correlation with retinal perfusion in the macula. The reduction of retinal vascular perfusion in diabetic people has been well documented in previous research [18, 19]. Our study further corroborates that FPG levels have a distinct and direct negative correlation with retinal perfusion. This suggests that controlling blood glucose levels is a crucial therapeutic measure to ameliorate retinal ischemia and hypoxia and to prevent the onset of diabetic retinopathy in diabetic people. Zhou,WJ et al. [20] found that FPG in non-diabetic people was also correlated with retinal SCP. However, in the non-diabetic group, the correlation for FPG was not significant, whereas serum total bilirubin and LDL concentration exhibited a clear positive correlation. Bilirubin is considered an important endogenous antioxidant [21]. Several studies have shown a protective association between high bilirubin levels and cardiovascular disease [22, 23] and coronary artery disease [24, 25]. A cross-sectional study reported that serum bilirubin levels may also have a protective effect against diabetic retinopathy due to its anticomplement and antioxidant stress-mediated properties [26–28]. However, no studies focusing on the relationship between total serum bilirubin levels and retinal microvascular changes in normal participants have been found. Elevated total bilirubin levels not only play a protective role in diabetic retinopathy, [28] but may also be resistant to the development of some ischemic and hypoxic diseases of the fundus in normal participants. This suggests that total serum bilirubin levels could be an important biomarker for identifying the degree of retinal microvascular perfusion in a non-diabetic population and may also laterally reflect the level of oxidative stress in the organism.

We conducted a comparative analysis of systemic and retinal vascular parameters in normal and diabetic groups. In line with the findings of previous studies [29–31], both PD and VD in the macular region of diabetic people in this study were significantly lower than in the non-diabetic group, suggesting early modifications of the microvascular network at the level of the superficial retinal layer. These reductions observed in end-vessel density and perfusion may be associated with hyperglycemia-induced vascular damage including the polyol pathway, advanced glycation end products accumulation, the protein kinase C pathway and the hexosamine pathway [32, 33]. In the multivariate analysis of FPG, concentration level of LDL-C and FPG was positively correlated, suggesting the promotion of diabetes by elevated LDL-C. More than two-thirds of people with type 2 diabetes are reported to have hypertension, the development of which coincides with the development of hyperglycemia. Many pathophysiological mechanisms underlie this association. Among these mechanisms, insulin resistance in the nitric oxide pathway; [34] the stimulatory effects of hyperinsulinemia on sympathetic drive, smooth muscle growth, and sodium retention; [35, 36] and the excitatory effects of hyperglycemia on the renin–angiotensin–aldosterone system seem plausible [37, 38]. In diabetic people, hypertension increases the risk of cardiovascular disease [39]. The FPG multivariable linear regression model also further confirmed that both PD and VD in the paracentral recess and whole macular region were relevant OCTA parameters. And elevated blood pressure and lipids are more likely to lead to hyperglycemia.

We also discovered that age had a significant impact on the retinal microvasculature in each region. Consistent with previous studies, [40–42] age was negatively correlated with macular PD and VD. Prior investigations [43] have demonstrated that deterioration of brain capillary flow and structure is a hallmark of aging. Although the mechanism is unclear, changes in retinal microvasculature with age may also be relevant. Few sex-related systemic factors or retinal vascular parameters were found in this study. The comparison between genders may not be significant due to the predominantly male people in this study.

We did not find other systemic factors with an independent relationship to retinal vascular density in this study. In agreement with previous studies, [20, 40] no linear relationship was found between parameters such as TC, UA, HDL, GPT and retinal VD. However, factors such as FPG, creatinine concentration and smoking history have also been reported [20] to have an effect on vascular parameters. Therefore, when analyzing the vascular parameters of fundus OCTA, all these systemic determinants should still be included to isolate their interactive effects.

This study has some limitations. First, because the participants in the original cohort study were not population-based, a selection bias might have occurred. Second, as a cross-sectional study, we were unable to identify the correlations found in the results as causal relationships. Besides, due to the lack of duration of diabetes, we were unable to analyze the effect on DR severity and retinal microvascular system. More prospective and rigorous studies are still needed to overcome these potential limitations. Nevertheless, there are some strengths of our study. Based on data and questionnaires from a 10-year follow-up, we have more comprehensive information about the physical condition of our large-sample participants. We included two parameters representing retinal vascular density, PD and VD, and were able to analyze the factors influencing retinal microvasculature in a multidimensional manner. In addition, we performed an exhaustive and rigorous statistical analysis. Each included parameter was carefully screened in a multivariate regression model to obtain more accurate results.

## Conclusion

In conclusion, decreased retinal VD and PD under OCTA assessment were associated with a high prevalence of diabetes and elevated fasting blood glucose concentration. Hypertension and hyperlipidemia are important risk factors for the development of atherosclerotic cardiovascular disease and diabetes but have no significant effect on retinal microvascular abnormalities. These data also suggest that OCTA can monitor early subclinical changes in retinal microvasculature and regular OCTA screening is beneficial for the diagnosis, classification and intervention of chronic systemic disease**.**

## Data Availability

The datasets generated and analyzed during the current study are available from the corresponding author on reasonable request.

## Abbreviations

OCTA: Optical coherence tomography angiography
DR: Diabetic retinopathy
NPDR: Non-proliferative diabetic retinopathy
PD: Perfusion density
VD: Vascular density
FAZ: Foveal avascular zone
SCP: Superficial capillary plexus
BP: Blood pressure
WC: Waist circumference
HC: Hip circumference
BMI: Body mass index
FPG: Fasting plasma glucose
HDL-C: High-density lipoprotein cholesterol
LDL-C: Low-density lipoprotein cholesterol
TG: Triglycerides
TC: Total cholesterol
UA: Uric acid
HsCRP: Hypersensitive C-reactive protein
GPT: Glutamate pyruvate transaminase
AL: Axial length
LT: Lens thickness
ACD: Anterior chamber depth
